# Exploring the role of cerebrospinal fluid as analyte in neurologic disorders

**DOI:** 10.2144/fsoa-2023-0006

**Published:** 2023-04-04

**Authors:** Sandeep C Pingle, Feng Lin, Misa S Anekoji, C Pawan K Patro, Souvik Datta, Lawrence D Jones, Santosh Kesari, Shashaanka Ashili

**Affiliations:** 1 CureScience Institute, 5820 Oberlin Drive #202, San Diego, CA 92121, USA; 2 Rhenix Lifesciences, 237 Vengal Rao Nagar, Hyderabad, TG, 500038, India; 3 Department of Translational Neurosciences, Saint John's Cancer Institute at Providence Saint John's Health Center & Pacific Neuroscience Institute, Santa Monica, CA 90404, USA

**Keywords:** biomarkers, cerebrospinal fluid, circulating cells, genomics, malignancy, neurodegenerative disorders, small molecules

## Abstract

The cerebrospinal fluid (CSF) is a clear ultrafiltrate of blood that envelopes and protects the central nervous system while regulating neuronal function through the maintenance of interstitial fluid homeostasis in the brain. Due to its anatomic location and physiological functions, the CSF can provide a reliable source of biomarkers for the diagnosis and treatment monitoring of different neurological diseases, including neurodegenerative diseases such as Alzheimer's disease, Parkinson's disease, amyotrophic lateral sclerosis, and primary and secondary brain malignancies. The incorporation of CSF biomarkers into the drug discovery and development can improve the efficiency of drug development and increase the chances of success. This review aims to consolidate the current use of CSF biomarkers in clinical practice and explore future perspectives for the field.

## Plain language summary

Cerebrospinal fluid (CSF) is a clear fluid that protects our brain and spinal cord, and can help diagnose and monitor neurological diseases like Alzheimer's and Parkinson's. Biomarkers in CSF are like clues that help doctors and researchers better understand these diseases. By using CSF biomarkers, doctors can diagnose and monitor patients more accurately, while researchers can develop more effective treatments. This review looks at how we use CSF biomarkers in medicine and how they might help us in the future. Better understanding of CSF biomarkers can improve the lives of people living with neurological diseases.

## Background

### Cerebrospinal fluid physiology

The cerebrospinal fluid (CSF) is a clear, colorless fluid that envelopes the central nervous system. The so-called CSF space is made up of intracerebral ventricles, subarachnoid spaces of the spine and brain, and the central spinal cord canal.

Normal healthy adults have approximately 150 ml of CSF that is distributed within the cranial and spinal subarachnoid spaces (∼125 ml), and ventricles (∼25 ml). CSF is secreted at an average rate of 20 ml/h or 400–600 ml/day, most of it by the choroid plexuses of the ventricles. The remainder of the CSF is secreted by the interstitium and the meninges. It maintains a pulsatile flow in a unidirectional rostrocaudal manner in the ventricular cavities, and in a multidirectional manner in the subarachnoid spaces. The CSF circulates through the intracerebral ventricles, cisterns, and subarachnoid space, and is absorbed (resorbed) at the level of arachnoid villi into the internal jugular venous system, and by cranial and spinal nerves, and the ependyma. The entire CSF volume is renewed 4–5-times every 24 h in adults. Interestingly, MRI studies have identified a notable circadian rhythmicity in CSF physiology; there is an approximately fourfold difference in the rate of CSF production between 6 p.m. (12 ml/h) and 2 a.m. (42 ml/h) [1].

### Composition of CSF

Physiologically, the CSF is an ultrafiltrate of blood secreted via the choroid plexuses. A multitude of studies have examined the various components of CSF, both in health and disease [2,3]. CSF comprises 99% water (compared with 92% water in plasma); the sodium, chloride, and magnesium concentrations are higher than those in plasma, whereas the potassium and calcium concentrations are lower than those found in plasma [4]. Specifically, the CSF concentrations of potassium (K^+^) are regulated stringently to ensure that any K^+^ lost from the ventricular system is replaced immediately; CSF K^+^ levels are maintained at a concentration of approximately 2.8 mM. In addition to the different ions, CSF also has cells, proteins, small molecules such as neurotransmitters and their metabolites, glucose, lactate, vitamins B1, B12, C, folate and b2-microglobulin, and nitric oxide secreted from the choroid plexus, as well as circulating DNA, RNA and microRNA.

### Functions of CSF

The main function of the CSF is to mechanically protect the brain and spinal cord. CSF passively transports to dural venous sinuses via arachnoid granulations to regulate brain volume and clear antigens [5]. In addition, it maintains homeostasis of the interstitial fluid of the brain parenchyma through electrolyte and acid-base balance, thereby regulating neuronal function. Furthermore, it provides a means of delivering nutrients to neurons and glia, and also removes metabolic waste products from the central nervous system [6]. Relatively recent scientific evidence points to the role of CSF in regulating the sleep-wake cycle through its effect on prostaglandin synthesis, specifically prostaglandin D2 [7].

### Clinical significance

Physiological CSF pressure varies between 10 and 15 mmHg in adults and 3 and 4 mmHg in infants [8]. Though this pressure is dynamic and varies with posture, physical activity, respiration and abdominal pressure, the average CSF pressure is relatively stable when the rate of CSF formation matches the rate of resorption, and when its circulation continues without any obstruction. A disequilibrium between formation and resorption can lead to an increase in CSF volume and pressure termed hydrocephalus [9,10]. Further, obstruction to normal circulation of the CSF can increase intracranial pressure proximal to the point of blockade.

**Figure 1.**
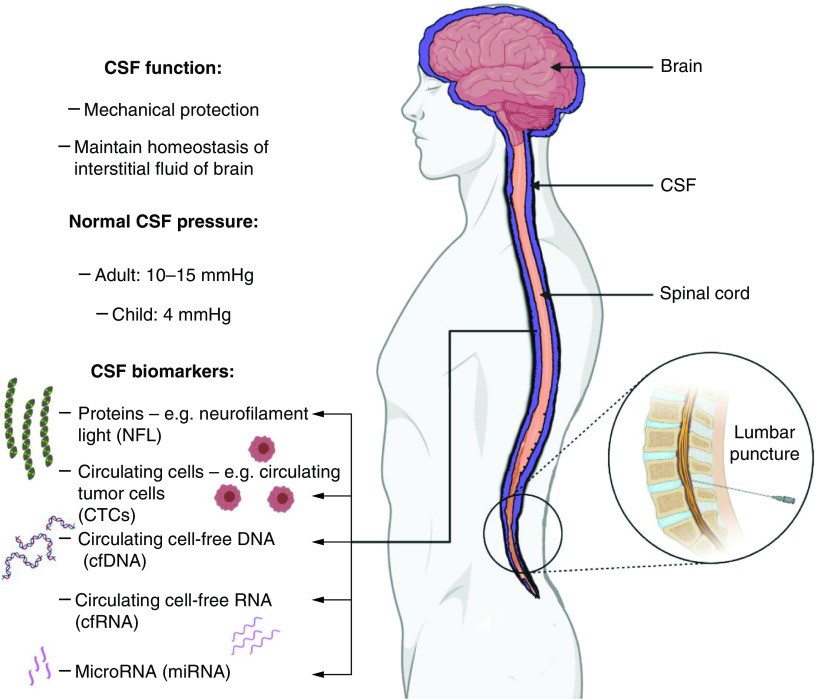
Overview of cerebrospinal fluid pathophysiology.

Apart from the clinical implications of such dysregulation, the CSF may serve as a reliable indicator of the health of the central nervous system. To this end, the CSF finds application as a valuable analyte to probe the brain and spinal cord in a minimally invasive manner. Specifically, the physical and molecular changes in the CSF may be valuable diagnostic and prognostic biomarkers for different neurological diseases [11]. This review will focus on CSF-based biomarkers and related clinical application in various neurological disorders. The physiology and biomarkers of CSF are summarized in Figure 1.

## CSF-based biomarkers

Different inflammatory or immune molecules including various proteins, other cell-free molecules and nucleic acids are secreted in the CSF. These can serve as quantifiable biomarkers in the CSF and include brain-derived proteins, exosomes, metabolites, small molecules, cell-free RNA, and cell-free DNA. These CSF-based biomarkers may find application in minimally invasive diagnosis and treatment monitoring of primary and secondary neurological diseases [12].

### Protein biomarkers

Proteins that exhibit altered expression levels in the CSF during the development and progression of neurological diseases can act as valuable disease-tracking biomarkers. Many synaptic and axonal proteins have been studied and explored as possible biomarkers, both in CSF and blood. Two well studied putative biomarkers are neurofilament light (NFL) polypeptide and neurogranin. NFL is a protein expressed in large, myelinated neurons. Its levels in the blood and CSF have been shown to increase following neurodegeneration [13]. A postsynaptic protein named neurogranin is expressed in dendritic spines. It is important in synaptic plasticity and an increase in circulating levels of neurogranin are typically associated with synaptic degeneration. Increased CSF levels of neurogranin have been demonstrated to be specific to Alzheimer's disease [14].

### Cytology: circulating cells

Circulating cells are cytologic biomarkers in the circulatory system and can be detected in blood or in fluids such as the CSF. These cells typically originate from malignancies, either primary tumors or metastases, and are termed as circulating tumor cells (CTCs). Most CTCs are rapidly destroyed in the blood circulation, which in turn limits their metastatic potential – only 2.5% of CTCs form micrometastases, whereas only 0.01% cells induce macrometastases [15]. CTCs in the CSF have potential diagnostic significance – they can be highly sensitive and accurate for cancer prognosis and measuring therapeutic efficacies. CTCs can detect the presence of malignancies earlier than traditional imaging techniques; it is a promising line of both diagnosis of brain metastasis progression and prognosis [16]. CTCs and their aggregates or emboli may invade CSF through different mechanisms that involve crossing the compromised blood brain barrier by blood and lymphatic system.

A promising emerging trend for CSF analysis is liquid biopsy, either using immunofluorescence, or fluorescence *in situ* hybridization (FISH) technologies [16]. However, FISH is currently not optimized for liquid biopsy and requires further research and development to elucidate whether this method is reliable for identification of CTCs. Other tools employed include integration of array comparative genomic hybridization (ACGH) analysis and other genomic techniques including deep sequencing [17,18]. Additionally, conventional flow cytometry analysis of CSF samples may prove useful for CTC analyses and diagnoses in CSF [16]. Other experimental approaches such as new generations of MRI, including phase-contrast MRI may enable quantitative measurements of CSF flow. However, this may pose problems with detection of relatively fast-moving single CTCs and particles due to slow time response [18,19]. The most advanced and promising method for detecting CTCs in the CSF is photoacoustic (PA) flow cytometry (PAFC) [20]. Additional studies are essential in understanding the correlative nature of CTCs with active disease and to establish the clinical utility of these biomarkers for both diagnosis and monitoring (progression, response to therapies, etc.).

### Genomics: circulating DNA, RNA, microRNA

Cell-free DNA: Cell-free DNA (cfDNA) in circulation, either in plasma or body fluids such as CSF, pleural fluid, or urine is a rich source of information on the physiological and pathological states of the individual. Though cfDNA in these body fluids is from different tissue origins, a detailed genomic analysis can differentiate and identify the source of origin of these molecules. cfDNA molecules have increasingly become promising biomarkers in the detection and monitoring of many diseases, mainly using next-generation sequencing for genome-wide analyses and single-base resolution. The analyses of cfDNA has facilitated blood-based diagnoses in place of tissue biopsies in the settings of prenatal and cancer care [21,22]. The existence of cfDNA in body fluids of healthy individuals is as a result of cellular apoptosis, whereas in pregnant women, the main source of fetal-derived cfDNA in maternal plasma is the placenta [23,24]. On the other hand, cfDNA in cancer patients is as a result of both apoptotic and necrotic events in tumors, leading to the presence of circulating tumor cfDNA or ctDNA. These DNA molecules are naturally fragmented and released into the circulation, and can be identified and characterized for molecular aberrations [25].

Cell-free RNA: Cell-free RNA (cfRNA) is a novel area in the field of circulating biomarkers that is also a challenging avenue in diagnosis and prognosis of diseases. Circulating *cfRNA* are gene transcripts that can be detected in the body fluids of cancer patients. In spite of the presence of nucleases in the circulation, *cfRNA* is surprisingly well protected from degradation, likely due to its packaging into exosomes [26]. *cfRNA* can be detected in body fluids using microarray technologies or reverse transcription quantitative real-time polymerase chain reaction (qRT-PCR) [26,27]. The benefit of *cfRNA* over cfDNA is that the former enables researchers and clinicians to detect fusion genes [28].

Micro RNA: MiRNAs are small endogenous mediators of RNA interference and key regulatory components of many biological processes. Many miRNAs are deregulated in cancers either as oncogenic (oncomirs) or as tumor suppressors. MiRNAs are found in the blood serum and other body fluids including CSF. MiRNAs have the potential to serve as biomarkers for diseases; cancer diagnostics using *miRNA* profile is an upcoming area of interest [29,30]. The detection of miRNA in CSF has raised the possibility of its use as biomarkers of neurologic diseases, including different types of brain cancer [31,32]. Other studies have shown miRNAs dysregulated in various neurodegenerative disorders; for example *miR-146a* and *miR-155* are upregulated in the CSF of patients with Alzheimer's disease [33].

### Epigenomics: methylated DNA

Epigenetics is the study of chemical modifications in the DNA, which regulates the gene expression or cellular phenotype associated with disease. Epigenetic modifications such as methylated DNA are epigenetic aspects that can be exploited to interrogate for specific CSF biomarkers associated with different diseases. The most common method is bisulfite treatment to distinguish between methylated and unmethylated DNA, which converts unmethylated cytosine in DNA to uracil. Further methylation-specific PCR can quantify DNA methylation limited to one genomic locus [34,35]. Many genomic loci can also be evaluated in parallel. Methylated DNA can have important implications and its analyses as biomarkers provides an additional tool for researchers and clinicians; for example, in gliomas, the most discussed epigenetic alteration is promoter hypermethylation of the gene for O6-methylguanine-DNA methyltransferase (MGMT) [12].

### Small moleclules

The CSF contains a range of small molecules that can be measured for diagnostic purposes. Neurotransmitters, such as dopamine, norepinephrine and epinephrine, along with their metabolites, can help diagnose certain neurological and psychiatric diseases, such as Parkinson's disease and depression [36,37]. Neopterin, a metabolite of guanosine triphosphate, can serve as an early biomarker of the cellular immune response, elevated levels in the CSF can indicate inflammation of the CNS [38]. Creatinine, a waste product of muscle metabolism, is normally filtered by the blood–brain barrier (BBB), and elevated levels in the CSF can be a sign of impaired BBB function [39]. Lactate, a metabolite from glucose, is typically present in low concentration in the CSF but can be increased in conditions such as cerebral ischemia, viral infections, brain tumors, and metabolic disorders [40,41]. Glucose levels in the CSF can also be measued to diagnose conditions such as meningitis or encephalitis, where levels are typically lower than normal [40].

Having reviewed the different types of biomarkers that can be detected in the CSF, the second half of this review will focus on CSF biomarkers for neurodegenerative diseases and brain malignancies.

## CSF as an analyte in diseases

Appropriately described by the term “CSF analytic brain” over 25 years ago, it has been clear for a long time that pathologies of the central nervous system are in most cases associated with changes in the CSF that may be detected through examination [42]. With increasing scientific evidence, it is now clear that owing to its anatomic location and physiological functions, CSF serves as an important tool in the diagnoses and prognoses of diseases of the nervous system [11]. It is recognized as a reliable source of biomarkers for many neurologic disorders, particularly neurodegenerative diseases such as Alzheimer's disease, Parkinson's disease, and amyotrophic lateral sclerosis. The biological fluid closest to the brain is CSF. Unlike plasma, CSF is not separated from the brain by the blood–brain barrier. Hence, proteins linked to brain-specific activities or to disease processes are more represented in the CSF than in other fluids or tissues [8]. However, it should be noted that all brain areas are not uniformly represented in CSF analysis, because based on anatomic location, some areas of the CNS contribute more to the CSF composition than others.

For CNS pathologies such as neurodegenerative disorders and brain malignancies, it is very difficult to obtain tissue-based biopsies for diagnosis; it is even more difficult to obtain multiple biopsies for longitudinal studies. It is in such cases where CSF-based diagnostics will play an important role. To this end, CSF and blood are being studied as potential analytes for many CNS disorders. Biomarkers have been identified, tested, and validated for cancer and for neurodegenerative diseases. For many CNS disorders such as neurodegenerative diseases, CSF provides a distinct advantage over blood owing in large part to its proximity to the brain parenchyma. Due to this physical proximity, proteins can be secreted from the brain extracellular space into the CSF, which can then be collected through spinal tap. Similarly, in the case of malignancies, nucleotides can be released into the CSF that can be detected by various novel techniques. CSF is routinely collected in the clinical setting, primarily for diagnoses of many brain disorders including CNS infections and degenerative diseases. Once collected, CSF analyses can help detect and characterize these secreted biomarkers biochemically. Please see Table 1 for a list of selected commercially available tests.

**Table 1.** List of commercial biomarker tests.

Commercial source	Available testing	Details
10X Genomics	Single cell multiomics	Single cell gene expression, epigenetics, immune profiling, transcript profiling
Adaptive Biotech	clonoSeq – NGS test	Measures minimal residual disease
Alacris Theranostics	Comprehensive molecular tumor analysis	NGS to seek out clinically actionable mutation
Biocept	CTC, ctDNA	Circulating nucleic acids, circulating tumor cells
Caris	Whole genome sequencing	Tumor profiling to assess DNA, RNA, and proteins
GRAIL	Multi-cancer detection	Galleri – early multi-cancer detection due to early detection
Guardant	Comprehensive Genome Profiling, Residual disease and recurrence monitoring	Liquid biopsy test to detect molecular response or changes in ctDNA levels
Isoplexis	Single Cell Proteomics	Proteomic suite for functional phenotyping; predict resistance to targeted therapeutics
Luminex	Multiplex testing (nucleic acids, proteins)	xMAP Technology - protein - and *nucleic* *acid*-based multiplex assays to simultaneously detect up to 500 targets

### CSF & Alzheimer's disease

Alzheimer's disease (AD) represents an unmet medical need owing to a severe lack of effective therapies. Many of the so-called disease-modifying drugs that looked promising have failed to make an impact on the lives of patients. To develop better therapeutic options for patients with AD, it is important to identify and characterize well-validated biomarkers for early detection and accurate diagnosis. This is because failure to successfully develop effective treatment options in part reflects on the inefficiency of current clinical trials that may not be targeting or enrolling the “appropriate” patient populations. There are numerous scientists currently looking at biomarkers in multiple biological fluids (such as CSF and blood) in combination with imaging and neuropsychological testing.

Genetically, AD is classified into two subtypes depending on the age of onset – early age of onset (before the age of 65 years and often in the late 40 s or early 50 s) labeled as early-onset Alzheimer's disease (EOAD) and comprising 1–5% cases; and disease developing after the age of 65 years classified as late-onset Alzheimer's disease (LOAD).

Beta-amyloid (Aβ) protein was identified to be present in plaques, while phosphorylated Tau aggregates were detected in tangles, in the brains of patients with AD [43,44]. While these research findings had implications for identifying new therapeutic targets for this disease, it also initiated a search for biomarkers, both for diagnosis and prognosis. Primarily, CSF was targeted as a source of biomarkers – proteins related to Alzheimer's disease were examined.

Extensive research in this area has so far identified a few CSF-based biomarkers. These include Aβ isoform Aβ1–42 (Aβ42), total Tau, phospho-Tau (p-Tau), and neuro filament light chain (NFL), measured by ELISA [45]. The main biomarker among these is the CSF levels of Aβ42 that have been demonstrated to have a sensitivity of 86% and specificity of 89% for AD. In addition, total Tau has 81% sensitivity and 91% specificity, while p-Tau has 81% sensitivity and 91% specificity in diagnosing AD [46]. In this regard, low CSF concentrations of Aβ42, along with high CSF concentrations of total Tau and/or p-Tau, when examined with imaging markers can indicate disease progression. In contrast, some studies have found the ratio of CSF levels of Aβ(1–42)/Aβ(1–40) to have stronger diagnostic accuracy for AD [47].

In some Alzheimer's cases, autoantibodies have been detected by ELISA against certain brain-derived proteins. One such autoantibody that was validated included the Aβ autoantibody in cognitively normal older population. Anti-Aβ42 autoantibodies were significantly higher in cognitively normal individuals when compared with age-matched Alzheimer's patients [48,49]. Based on these data, there are ongoing studies examining the feasibility of anti-Aβ antibodies as therapeutic agents against AD.

### CSF & Parkinson's disease

Parkinson's disease (PD) is a progressive neurodegenerative disease that affects ∼1% of the adults over 60 years of age [50]. The primary pathophysiology of this disease is due to loss of dopamingergic neurons, which leads to motor and non-motor features. The main clinical signs of this disease include bradykinesia, tremor, rigidity or postural instability [51]. Scientific efforts have focused on improving early diagnosis by utilizing different modalities including imaging and biochemical biomarkers. Characterization of biomarkers remains in its early stages and both blood and CSF are being used as sources. Not only will good biomarkers help in diagnoses but will also facilitate drug development.

α-Synuclein has been shown to aggregate and is found in Lewy bodies in patients with PD. This has made α-Synuclein a prime target for biomarker studies in both CSF and blood. In a few previous studies using large patient cohorts, it was observed that there were lower levels of α-synuclein in the CSF of patients with PD when compared with healthy controls [52–55]. The levels of α-synuclein were primarily measured by ELISA [53,54]. Sensitivity and specificity were caculated using a classification table, which revealed high specificity (80–100%) but low sensitivity (20–70%) [54]. However, more recently, these data were challenged by some additional studies that looked at CSF levels of α-Synuclein in patients with PD that failed to show any correlation between disease severity and CSF levels [56], even increased total plasma or serum α-Synuclein levels were found in early stages of the PD disease [57,58]. The levels of α-Synuclein can vary between control and patient groups in different research reports, and statistical analyses are used to determine whether these differences are significant. However, inconsistent results may arise due to the use of different assays, limited numbers of patients, and inadequate control of important variables, and sampling from different disease stages in these reports. Further research is needed to evaluate the role of α-Synuclein in PD in different stages and characterize it further as a biomarker that can have clinical utility.

In addition to α-Synuclein, studies have demonstrated that familial PD is associated with mutations in DJ-1 (PARK7) [52]. Importantly, DJ-1 has been detected in both CSF and plasma samples by ELISA. This represents a promising lead as a biomarker in familial PD and is being studied further. Dopamine has been shown to decrease in individuals with PD, the decrease is thought to contribute to the motor symptoms of the disease [36,37].

An interesting finding from a recent study highlights biomarkers that are originally being studied in the context of AD. This study from a cohort of untreated PD patients collected sequential samples till patients received levodopa therapy. They found that following levodopa treatment, those patients who had a higher p-Tau and a p-Tau/Aβ42 ratio in the CSF developed cognitive decline subsequently [59]. This finding needs to be validated further but if true, will be a biomarker for prediction of therapeutic effectiveness. Biomarkers like these will be invaluable in clinical studies to select appropriate patient cohorts for various emerging therapies.

### CSF & amyotrophic lateral sclerosis

Amyotrophic lateral sclerosis is a paralytic disease characterized by progressive motor neuron degeneration in the brain and spinal cord. The pathologic hallmark of this disease is motor neuron death in the motor cortex and spinal cord. The clinical presentation of ALS is heterogeneous, which makes clinical diagnosis difficult. Moreover, there is no diagnostic test that can definitively confirm or rule out ALS. Though the exact pathophysiological mechanisms underlying ALS are not clear, multiple genes have been implicated by gene mapping and DNA analyses [60]. Currently, there is no effective therapy with definitive clinical benefit. There are two drugs that are used for ALS therapy (riluzole and edaravone), but these drugs only provide limited survival benefit. While targeted therapies are being developed against ALS, biomarkers will become central to early diagnoses, selection of appropriate patient groups, and monitoring effectiveness of therapy.

Numerous proteins in the CSF and blood are being evaluated as potential biomarkers for ALS [61,62]. NFL and phosphorylated heavy chain (pNFH) proteins are a group of neurofilament proteins that may serve as diagnostic and prognostic biomarkers for ALS, measured by ELISA. There are reports that have demonstrated increases in the levels of pNFH in the CSF of ALS patients when compared with both healthy controls and neurologic disease controls [63,64]. Importantly, data from several studies indicates that pNFH levels in CSF and blood correlate with the rate of disease progression and survival in ALS [65,66]. In addition, another study evaluated many commonly used ALS biomarker candidates in clinical ALS samples from six European centers. This multicenter sample-collection approach was taken to eliminate the variability associated with different laboratories. Using this approach, data obtained demonstrated that levels of pNFH in the CSF were significantly different between ALS and control cases in all centers [56,67]. Thus, pNFH represents a promising biomarker candidate for clinical translation.

Apart from neurofilament proteins, *TARDBP* (*TAR DNA-binding protein*) gene is mutated in some cases of familial ALS, TDP-43 (TAR DNA-binding protein-43) is a core component of cytoplasmic inclusions in ALS and FTLD. Hence CSF levels of TDP-43 are also being studied to evaluate its utility as a biomarker for ALS. Using antibody-based approaches, TDP-43 was found to have increased levels in ALS patients; interestingly, lower levels of TDP-43 were associated with worse prognosis and reduced survival [68].

Based on the inflammatory responses seen in ALS, different mediators of inflammation are also being studies in both CSF and blood, such as IL-8, wide-range C-reactive protein, etc [69–71]. Based on recent studies on metabolomics, it appears that disruption of metabolic pathways may drive some of the pathogenesis of ALS. To this end, metabolomic biomarkers are also being evaluated and profiled in plasma and CSF samples [72–74]. All of these are promising avenues for novel biomarkers and need to be explored further.

### CSF & brain malignancies

Currently, brain malignancies are challenging to both diagnose and treat, and are associated with high rates of mortality and morbidity [75]. Especially challenging is the aspect of monitoring patients for response to therapies, due to the risks associated with brain biopsies, and the low specificity and sensitivity of other noninvasive modalities including CSF analyses, cytology, and imaging. Hence there is an urgent unmet need to develop and validate biomarkers for brain malignancies. Blood-based biomarkers have been valuable and have found application in the diagnosis and monitoring of various types of peripheral cancers. However, they have proven suboptimal in malignancies involving the CNS, largely due to the presence of the blood–brain barrier [76,77].

In contrast, the physical proximity makes CSF a suitable analyte for biomarkers in brain malignancies. Moreover, CSF can be accessed readily and relatively non-invasively (via lumbar punctures) for longitudinal disease monitoring during and after therapy. While cytological analysis of CSF has low sensitivity, it is also non-quantitative [78]. Hence with regards to accuracy and reliability, CSF-based biomarkers such as ctDNA, microRNAs and metabolites may be the solution to accurate and minimally invasive assessment of brain malignancies [76,79–83].

CSF-based biomarkers can be used for early diagnosis of brain malignancies, early identification of recurrence, monitoring therapeutic responses, and personalized medicine strategies for targeted therapies. There are two main modalities for CSF biomarkers: ctDNA and miRNA.

#### CSF ctDNA

Circulating tumor DNA that is shed from the tumor can be detected in the CSF. Recent studies have demonstrated that the detection rate of ctDNA in CSF is higher than that in tumor cells by next generation sequencing [84] and droplet-digital PCR (ddPCR) [85,86]. Furthermore, ctDNA detection rates are comparable to those in other body fluids tested [87]. Detection of specific mutations and their relative levels in ctDNA can be achieved through digital droplet PCR (ddPCR), BEAMing, and next-generation sequencing. This is an important point, particularly since recent therapeutic advances have shifted from histopathological tumor type-based therapy to genomics-based therapy. The latter is dependent on accurate genomic characterization of the tumor, which requires tumor tissue for genetic analyses. However, ctDNA can act as a surrogate for tumor tissue to detect and monitor tumor mutational status prior to, during, and following targeted therapies. The main advantages of the ctDNA approach are that it can overcome the challenge due to temporal and spatial intratumor heterogeneity that can be missed in tissue biopsies. Furthermore, it can provide access to tumor DNA in cases where the tumor is difficult to surgically access. Besides, it is a relatively safer, cheaper, and less invasive option to interrogate the tumor profile.

ctDNA detection in the CSF has been demonstrated in both primary brain malignancies and in metastatic cancers; these analyses can be either restricted to a single gene or can involve a panel of multiple genes depending on the specific mutations in the tumor under consideration [88]. A myriad of studies has examined ctDNA in different CNS malignancies [89–97].

While this is an extremely powerful tool in detection and monitoring of brain malignancies, there are some significant limitations as well, to ctDNA-based CSF biomarkers, primarily the lack of sensitivity in case of some malignancies. The location of the tumor, mainly its proximity to the CSF may be an important factor that drives the levels of ctDNA in the CSF, particularly in the case of primary CNS tumors [75,87].

#### CSF miRNA

Many studies that have demonstrated that miRNAs can function as oncogenes (oncomirs) and/or tumor suppressors; significantly, dysfunctional expression of miRNAs is a common feature of many types of cancer [98,99]. Importantly, miRNAs are secreted in membrane vesicles (exosomes), blood serum, and other body fluids, including the CSF, which suggests a possibility of using miRNAs as potential biomarkers of neurologic diseases [29,30,100–105].

Other biomarkers such as tumor markers CEA, CA-125, and AFP can be produced by the brain tumors and detected in the CSF. Additionally, genetic mutations in *EGFR*, *IDH1/2* and *TP53* genes have also been associated with brain cancers and can be detected in the CSF. A recent review has highlighted the significance of these biomarkers [84].

The presence of these biomarkers in the CSF can aid in the diagnosis of brain cancer, facilitate monitoring of disease progression, and inform treatment decisions. Nevertheless, the interpretation of CSF biomarkers should be performed in conjunction with other clinical and radiographic findings, as well as histopathological analysis of tissue samples.

## Conclusion

Rigorous scientific approaches are crucial to identifying, characterizing, and validating CSF-based biomarkers for clinical use. It is essential to demonstrate their sensitivity and specificity before they can be applied in the clinical neurological setting. The confluence of various -omics technologies will undoubtedly facilitate and drive the development of CSF-based biomarkers and their clinical translation to address the various unmet needs in this challenging clinical space.

## Future perspective

CSF biomarkers should not be limited to the clinic but should also be incorporated into the drug discovery and development process early on to make drug development more efficient. Using appropriate biomarkers in clinical trial settings will improve chances of success through patient population stratification, identification of patient subpopulations most likely to respond, appropriate dose selection, demonstration of drug efficacy, and shortening the length of clinical trials. CSF biomarker-driven clinical strategies will make clinical trials time and cost efficient in both early and late stages.

**Executive summary**

**Background**

- CSF serves as a protective and regulatory fluid for the central nervous system, aiding in diagnosis and prognosis of neurological diseases.

**CSF-based biomarkers**

- CSF is a reliable source of biomarkers for neurodegenerative diseases and brain malignancies.
- CSF-based biomarkers can reflect pathophysiological changes related to a disease and aid in diagnosis and monitoring.

**CSF as an analyte in diseases**

- Biomarkers in CSF can be proteins, *nucleic acids*, or metabolites and can diagnose diseases such as Alzheimer's, Parkinson's and brain malignancies.
- Demonstrating sensitivity and specificity of CSF biomarkers is crucial for clinical translation, and innovative technologies can facilitate their development and validation.

**Future perspective**

- Incorporating CSF-based biomarkers into drug development can enhance target identification and drug efficacy optimization, leading to improved success rates and efficiency.
